# Correction: Chen et al. Hemodynamic Effects of Subaortic Stenosis on Blood Flow Characteristics of a Mechanical Heart Valve Based on OpenFOAM Simulation. *Bioengineering* 2023, *10*, 312

**DOI:** 10.3390/bioengineering11050495

**Published:** 2024-05-16

**Authors:** Aolin Chen, Adi Azriff Basri, Norzian Bin Ismail, Kamarul Arifin Ahmad

**Affiliations:** 1 Department of Mechanical Engineering, Faculty of Engineering, University Putra Malaysia, Serdang 43400, Selangor, Malaysia; 2 Department of Aerospace Engineering, Faculty of Engineering, University Putra Malaysia, Serdang 43400, Selangor, Malaysia; 3 Department of Medicine, Faculty of Medicine and Health Sciences, University Putra Malaysia, Serdang 43400, Selangor, Malaysia

In the original publication [1], there was a mistake in relation to the cited Reference [16] (Vickers, N.J., 2017 [2]), which has been replaced by the work of Guivier et al., 2007 [3], and the order of references remains the same.

The authors state that the scientific conclusions are unaffected. This correction was approved by the Academic Editor. The original publication has also been updated.
